# Association Between Neutrophil-to-Lymphocyte Ratio (NLR) and Platelet-to-Lymphocyte Ratio (PLR) With Diabetic Retinopathy in Type 2 Diabetic Patients

**DOI:** 10.7759/cureus.48581

**Published:** 2023-11-09

**Authors:** Ana Maria Dascalu, Adriana Georgescu, Andreea Cristina Costea, Laura Tribus, Alae El Youssoufi, Dragos Serban, Andreea Letitia Arsene, Daniela Stana, Cristina Alexandrescu, Bogdan Mihai Cristea, Denisa Tanasescu, Anca Bobirca, Crenguta Serboiu, Catalin Alius, Dan Georgian Bratu

**Affiliations:** 1 Faculty of Medicine, Carol Davila University of Medicine and Pharmacy, Bucharest, ROU; 2 Doctoral School, Carol Davila University of Medicine and Pharmacy, Bucharest, ROU; 3 Nephrology and Dialysis, Diaverum Clinic, Constanta, ROU; 4 Faculty of Oral Medicine, Departement of Gastroenterology, Carol Davila University of Medicine and Pharmacy, Bucharest, ROU; 5 Faculty of Pharmacy, Carol Davila University of Medicine and Pharmacy, Bucharest, ROU; 6 Ophthalmology, Emergency University Hospital Bucharest, Bucharest, ROU; 7 Department of Nursing and Dentistry, Lucian Blaga University of Sibiu, Sibiu, ROU; 8 Department of Clinic Surgery, Lucian Blaga University of Sibiu, Sibiu, ROU

**Keywords:** prognostic model, platelet-to-lymphocyte ratio (plr), neutrophil-to-lymphocyte ratio (nlr), biomarkers, diabetic retinopathy

## Abstract

Diabetic retinopathy (DR) is one of the main causes of blindness worldwide, but an effective screening is challenging due to limited available retina specialists. Finding novel biomarkers could help clinical decision in prioritizing ophthalmological consultation in patients at risk of developing severe DR. This study aims to investigate the association between neutrophil-to-lymphocyte ratio (NLR), platelet-to-lymphocyte ratio (PLR), and the presence and severity of DR in patients with T2DM. A retrospective study was performed on 90 patients with T2DM admitted in the Ophthalmology Clinic, Emergency University Hospital Bucharest in Bucharest, Romania, between March 2022 and March 2023, for routine cataract surgery. The cases were divided into three groups according to the severity of DR: no DR (noDR), non-proliferative diabetic retinopathy (NPDR), and proliferative DR (PDR) groups. NLR values raised significantly in the PDR group, no DR group (p = 0.003), and NPDR group (p = 0.026), while PLR values did not differ statistically significant among the groups (p = 0.059). No difference in terms of age, sex, HbA1C, and comorbidities were observed. In the multivariate analysis, the NLR (OR = 2.01, [1.29; 3.14], p = 0.0019) and diabetic nephropathy (OR = 3.84, [1.23; 11.98], p = 0.0203) were associated with higher rates of PDR. NLR may be a promising tool in the risk stratification of T2DM patients with DR.

## Introduction

Diabetes mellitus (DM) is a major health problem globally, accounting for 537 million patients worldwide and with an expected ascendant trend, reaching 700 million by 2045 [1,2]. For type 2 diabetes mellitus (T2DM), which accounts for approximately 90% of the total, this rising trend can be attributed to aging, rapid increase in urbanization, and obesogenic environments [1].

Diabetic retinopathy (DR) is a common microvascular complication that affects individuals with type 2 diabetes mellitus (T2DM), leading to significant visual impairment and blindness worldwide. The prevalence of DR in diabetic patients is approximately 34.6% in T2DM patients, with vision-threatening DR present in 12%, making it a public health concern [3,4]. Several risk factors contribute to the development and progression of DR, including the history of diabetes, long uncontrolled hyperglycemia, arterial hypertension, dyslipidemia, chronic inflammation, and altered immune response. The early detection and diagnosis of DR are crucial for the timely intervention and prevention of vision loss. Despite its high prevalence and severe impact on vision, the early detection of DR remains a challenge, emphasizing the need for reliable predictive biomarkers.

The World Health Organization (WHO) Universal Eye Health: A Global Action Plan 2014-2019 outlines the need to achieve a reduction in the prevalence of avoidable visual impairment and blindness, including that related to diabetes, which is currently among the five most common causes of both moderate or severe visual impairment and blindness [5]. DR is listed as a priority eye disease in the 2030 IN SIGHT strategy [6]. However, an effective screening may be challenging due to limited available retina specialists.

In recent years, there has been a growing interest in exploring novel biomarkers that can provide insights into the pathogenesis and progression of DR. The neutrophil-to-lymphocyte ratio (NLR) and platelet-to-lymphocyte ratio (PLR) have emerged as potential markers of systemic inflammation and immune imbalance. The NLR reflects the balance between pro-inflammatory neutrophils and anti-inflammatory lymphocytes, while the PLR represents the interplay between platelets and lymphocytes [7,8].

The association between NLR, PLR, and various diseases has been extensively investigated in the medical field. Elevated NLR and PLR values were found to be predictors for adverse outcomes in cardiovascular diseases, malignancies, and chronic inflammatory disorders [7-10]. However, limited studies have explored the role of NLR and PLR in the context of DR in patients with T2DM. Understanding the potential association between NLR, PLR, and DR holds promise in providing valuable clinical information for risk stratification, early detection, and effective management of DR. Moreover, unraveling the underlying mechanisms linking these ratios with the pathophysiology of DR could contribute to the development of targeted screening and therapeutic interventions.

Therefore, this study aims to investigate the association between NLR, PLR, and the presence and severity of DR in patients with T2DM.

## Materials and methods

A retrospective comparative study was performed on 90 patients with T2DM admitted in the Ophthalmology Clinic, Emergency University Hospital Bucharest in Bucharest, Romania, between March 2022 and March 2023, for routine cataract surgery. The patients were included in three groups: no diabetic retinopathy (noDR, 30 patients), non-proliferative DR (NPDR, 30 patients), and proliferative DR (PDR, 30 patients). The inclusion criteria consisted of patients with age > 18 years, with previously diagnosed T2DM, and with a duration of DM of more than five years.

The exclusion criteria consisted of the presence of hematologic, oncologic, inflammatory, or infectious pathologies that might interfere with the dynamics of NLR and PLR. Moreover, we excluded patients with any retinal disorder that may interfere with the correct assessment of the progression of diabetic retinopathy, such as retinal detachment, inherited retinal diseases, associated chorioretinitis active or scarring, uveitis, central areolar choroid dystrophy, age-related macular degeneration, and advanced senile cataract.

The diagnosis and grading of DR was done by a comprehensive eye assessment, which includes a medical history, visual acuity testing, and a dilated fundus examination and optical coherence tomography (OCT) examination of the macula. Each patient was classified according to the International Classification of Diabetic Retinopathy (ICDR) severity scale as no retinopathy (no DR), non-proliferative DR (NDR), or proliferative DR (PDR). In order to minimize the risk of bias, DR was independently graded by two retina specialists in our clinic. Any disagreement was solved by discussions. All social distancing and frequent disinfecting requirements related to the COVID-19 pandemic were strictly respected [11].

All patients underwent a blood test for complete blood count with differentials, blood sugar, HbA1C, serum urea, and creatinine. NLR and PLR were calculated as the ratio of the total neutrophil count/total lymphocyte count and total platelet count/total lymphocyte count, respectively, measured from the same blood test, using automated hematology analyzing devices, and expressed in × 103 cells/μl.

Data processing

All data were included in an Excel table. Group comparability was assessed for the noDR, NPDR, and PDR groups in terms of age, sex, associated comorbidities, and biological data. The biomarkers under study included glycemia, HbA1C, neutrophils, lymphocytes, monocytes, thrombocytes, urea, creatinine, NLR, and PLR.

Numeric variables were expressed as mean (±standard deviation (SD)) and discrete outcomes as absolute and relative (%) frequencies. Normality and hetereoskedasticity of continuous data were assessed with Shapiro-Wilk and Levene’s test, respectively. Continuous outcomes were compared with analysis of variance (ANOVA), Welch's ANOVA, or Kruskal-Wallis tests according to the data distribution. Discrete outcomes were compared with chi-squared or Fisher’s exact test accordingly. The alpha risk was set to 5% and two-tailed tests were used. A regression analysis was then performed for the predictive factors for DR and PDR in the study group. Statistical analysis was performed with EasyMedStat (version 3.30; www.easymedstat.com).

## Results

A total of 90 patients, aged between 36 and 77 years, were included in the study. There were no differences in age and sex distribution among the three groups. The comparative characteristics are presented in Table 1.

**Table 1:** Comparative data of the patients included in the noDR, NPDR, and PDR groups *statistically significant value, p<0.05 noDR: no diabetic retinopathy; NPDR: non-proliferative DR; PDR: proliferative DR; NLR: neutrophil-to-lymphocyte ratio; PLR: platelet-to-lymphocyte ratio; SD: standard deviation; HbA1C: hemoglobin A1C

Variable	noDR group	NPDR group	PDR group	p-value
	N = 30	N = 30	N = 30	
Age (years, mean±DS)	66.9 (± 5.76)	66.4 (± 6.07)	62.0 (± 10.59)	0.088
Male sex (n, %)	12 (40.0%)	11 (36.67%)	12 (40.0%)	0.312
Ischemic cardiac disease (n, %)	2 (6.67%)	1 (3.33%)	6 (20.0%)	0.136
Arterial hypertension (n, %)	10 (33.3%)	6 (20.0%)	9 (30.0%)	0.437
Glycemia (mg/dL, mean±SD)	142.37 (± 38.95)	186.83 (± 84.89)	160.5 (± 52.43)	0.117
HbA1C (%, mean±SD)	7.01 (± 0.972)	7.33 (± 1.59)	7.25 (± 0.978)	0.467
Diabetic nephropathy (n,%)	3 (10.0%)	5 (16.67%)	12 (40.0%)	0.013*
Neutrophils (cells x10^3^/mmc, mean±SD)	4.69 (± 1.47)	4.73 (± 1.24)	5.27 (± 1.58)	0.157
Lymphocytes (cells x10^3^/mmc, mean±SD)	2.23 (± 0.735)	2.17 (± 0.73)	1.84 (± 0.894)	0.073
Monocytes (cells x10^3^/mmc, mean±SD)	0.522 (± 0.185)	0.538 (± 0.179)	0.638 (± 0.154)	0.009*
Thrombocytes (cells x10^3^/mmc, mean±SD)	229.69 (± 39.67)	258.67 (± 45.58)	242.82 (± 65.27)	0.04*
Urea (mg/dL, mean±SD)	42.97 (± 15.97)	50.9 (± 20.87)	54.82 (± 29.83)	0.19
Creatinine (mg/dL, mean±DS	0.865 (± 0.215)	0.97 (± 0.323)	1.3 (± 1.03)	0.036*
NLR (mean±DS)	2.28 (± 0.932)	2.44 (± 0.991)	3.51 (± 1.91)	0.008*
PLR (mean±DS)	113.49 (± 40.53)	126.19 (± 27.08)	165.67 (± 95.86)	0.059

**Figure 1:**
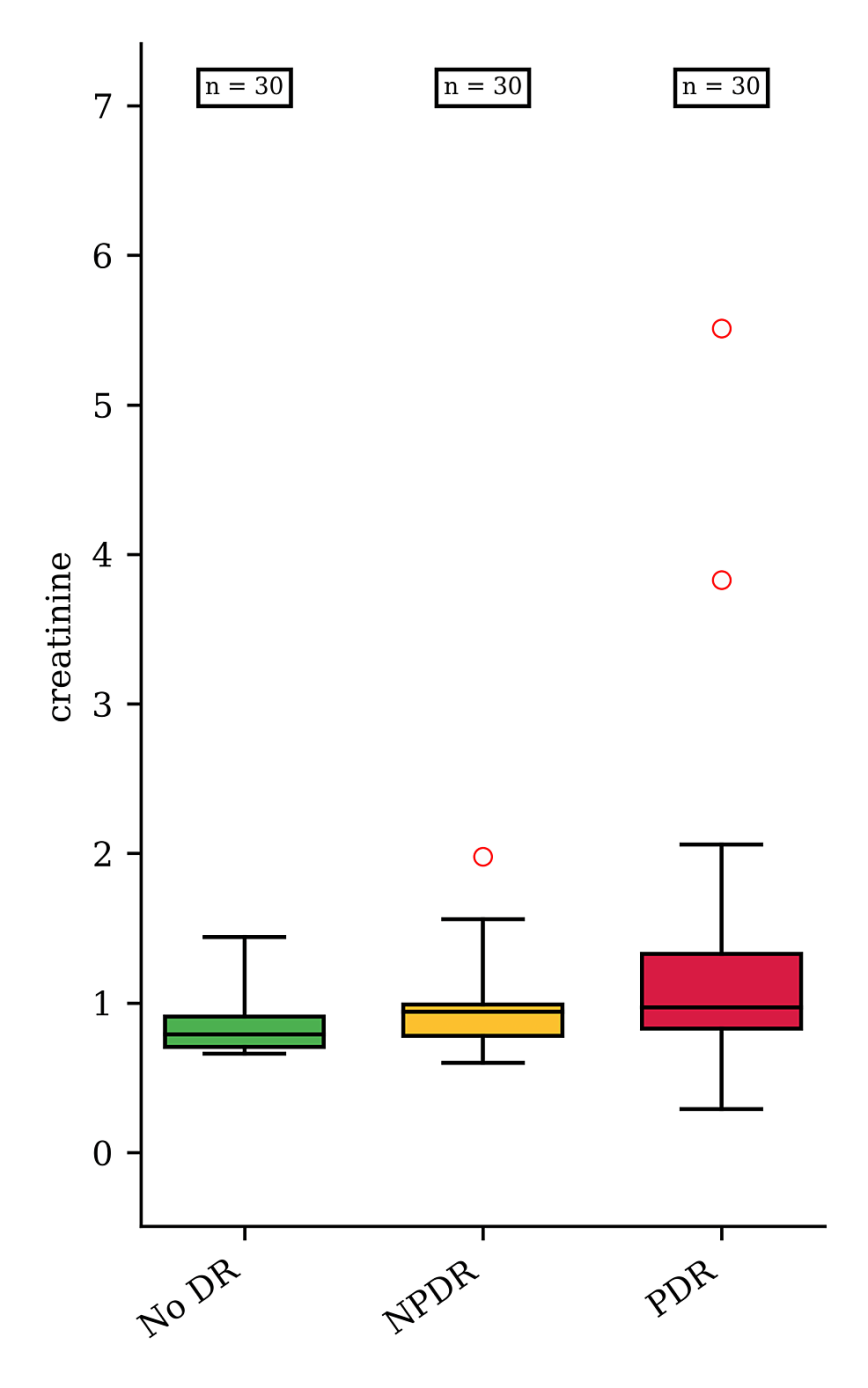
Serum creatinine in the study groups The median values of creatinine were 0.79 (interquartile range (IQR) 0.21), 0.94 (IQR 0.21), and 0.97 (IQR 0.5) in patients with no DR, NPDR, and PDR (p=0.036), respectively. Pairwise analyses revealed differences for PDR vs. NoDR (p = 0.01). noDR: no diabetic retinopathy; NPDR: non-proliferative DR; PDR: proliferative DR

Among the associated comorbidities, we found that diabetic nephropathy was encountered more frequently in the PDR group (p = 0.013), when compared to the noDR and NPDR groups, supporting the correlation between microvascular changes in T2DM in both the kidney and eyes. Moreover, the pairwise analysis showed that creatinine levels were higher in the PDR group vs. the noDR group (p = 0.01; Figure 1).

**Figure 2:**
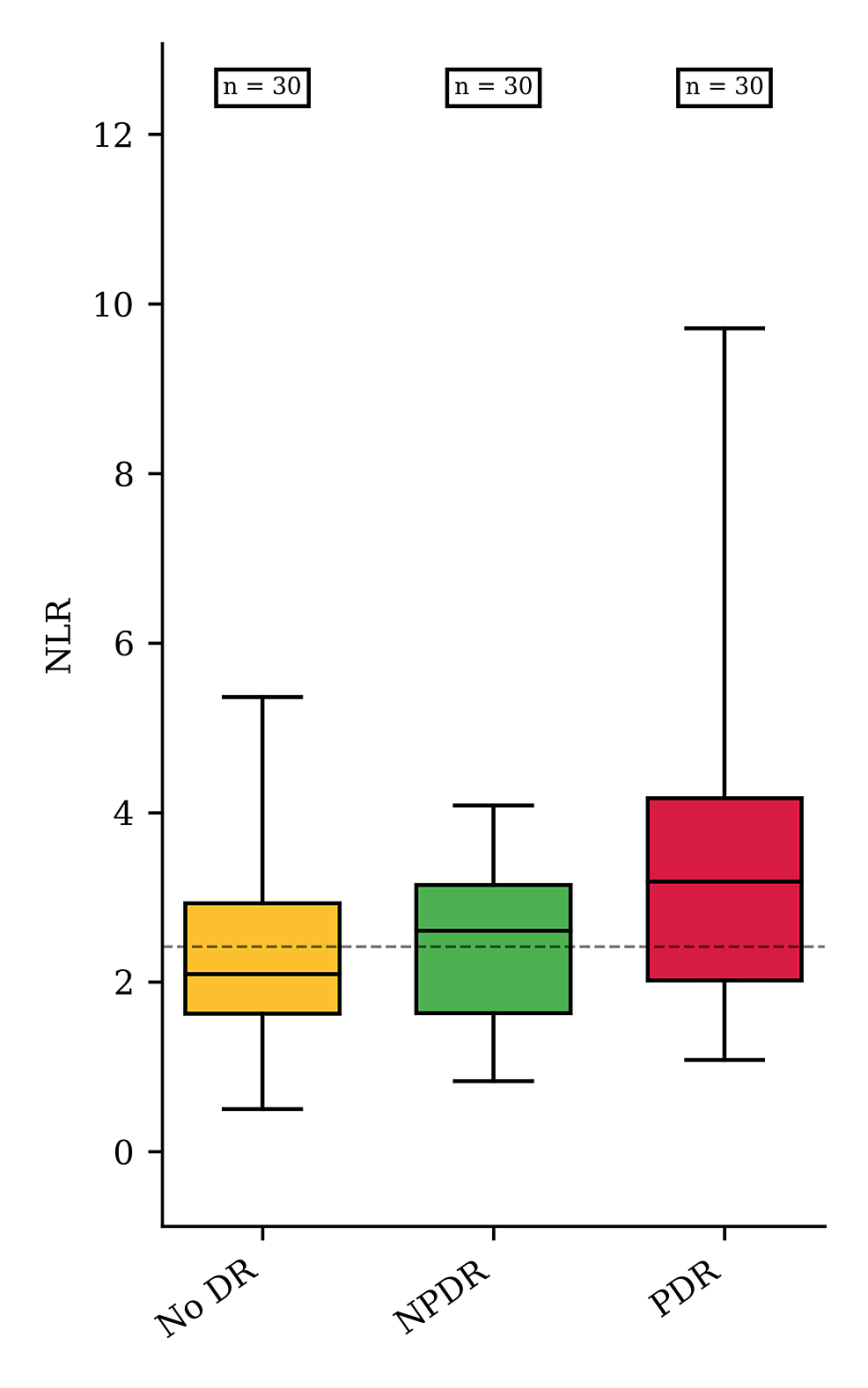
NLR values for the noDR, NPDR, and PDR groups The median values of NLR were 2.09 (interquartile range (IQR) 1.31), 2.6 (IQR 1.51), and 3.19 (IQR 2.15) in patients for the noDR, NPDR, and PDR groups (p=0.008), respectively. Pairwise analyses revealed differences for PDR vs. NoDR (p = 0.003) and PDR vs. NPDR (p = 0.026). noDR: no diabetic retinopathy; NPDR: non-proliferative DR; PDR: proliferative DR

While there were no differences in the total neutrophil count and lymphocyte count among the three groups, we encountered a significant rise in the NLR values from the noDR to PDR groups. Normality and heteroskedasticity of data were assessed with the Shapiro-Wilk test and Levene’s test. The difference between NLR according to the modalities of DR type was assessed with the Kruskal-Wallis. If the null hypothesis of Kruskal-Wallis test was rejected, post-hoc pairwise analyses were performed with the Dunn-Bonferoni test. The alpha risk was set to 5% (α = 0.05; Figure 2).

**Figure 3:**
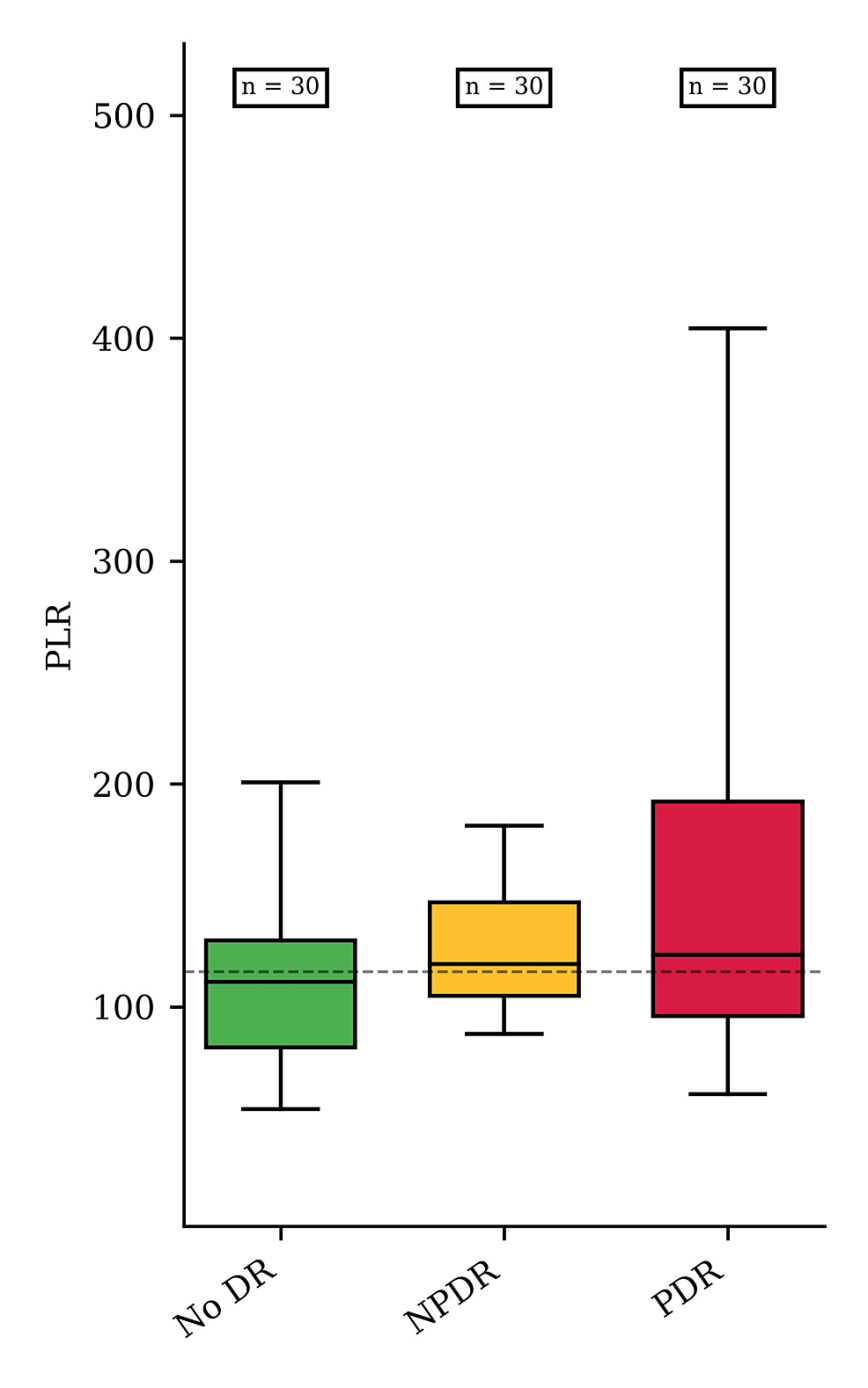
PLR values for the noDR, NPDR, and PDR groups The median values of PLR were 111.12 (interquartile range (IQR) 48.11), 119.12 (IQR 41.79), and 123.2 (IQR 96.3) in patients from the noDR, NPDR, and PDR groups (p = 0.059). noDR: no diabetic retinopathy; NPDR: non-proliferative DR; PDR: proliferative DR; PLR: platelet-to-lymphocyte ratio

The PLR increased with the stage of DR in the study group, with median values of the PLR raised from the noDR group (111.12) to the PDR group (123.1). However, the differences did not meet the statistical significance of p < 0.05 (Figure 3).

The median values of monocytes were respectively 0.5 (interquartile range (IQR) 0.2), 0.5 (IQR 0.2), and 0.61 (IQR 0.28) in patients included in the noDR, NPDR, and PDR groups (p = 0.009). The pairwise analyses revealed differences between the PDR and noDR groups (p = 0.01) and between the PDR and NPDR groups (p = 0.007), suggesting that the total monocyte value might be a biomarker for PDR in T2DM.

Furthermore, we analyzed the predictive value for the NLR and PLR for DR (any grade) and for PDR based on the data provided by the study group. A logistic regression analysis was performed to assess the relation between DR and the explanatory variables: NLR, PLR, monocytes, thrombocytes, and creatinine. Data were checked for multicollinearity with the Belsley-Kuh-Welsch technique. Heteroskedasticity and normality of residuals were assessed respectively by the Breusch-Pagan test and Shapiro-Wilk test. A p-value < 0.05 was considered statistically significant. The correlations were expressed as odd ratios (OR) in Table 2.

**Table 2:** Logistic regression for the dependent variables for DR *statistically significant (p<0.05) DR: diabetic retinopathy; NLR: neutrophil-to-lymphocyte ratio; PLR: platelet-to-lymphocyte ratio; HbA1C: hemoglobin A1C

Variable	Odds ratio [confidence interval]	p-value
NLR	1.55 [1.01; 2.38]	0.044*
PLR	1.01 [1; 1.02]	0.0342*
Monocytes	9.26 [0.638; 134.21]	0.103
Thrombocytes	1.01 [0.999; 1.02]	0.0742
Creatinine	5.05 [0.934; 27.32]	0.0601
Diabetic nephropathy	3.56 [0.952; 13.3]	0.0592
HbA1c	1.25 [0.808; 1.93]	0.317

In the univariate analysis, NLR (OR=1.55, [1.01; 2.38], p= 0.044) and PLR (OR: 1.01 [1; 1.02], p=0.0342) were associated with higher rates of DR, while monocytes, thrombocytes, HBA1C, creatinine values, and diabetic nephropathy were not associated with the risk of DR.

Moreover, the median NLR was respectively 2.89 (Q1 1.85; Q3 3.72) and 2.09 (Q1 1.62; Q3 2.93) for patients with and without DR (difference: 0.8, p=0.037), while the mean NLR was respectively 3.32 (±1.49) and 2.33 (±0.93) for patients with and without PDR (difference: -0.99 [-1.59; -0.39], p=0.002). The median PLR was respectively 123.08 (Q1 103.01; Q3 172.4) and 111.12 (Q1 81.72; Q3 129.83) for patients with and without DR (difference: 11.96 , p=0.026) and respectively 123.33 (Q1 96.32; Q3 188.53) and 113.93 (Q1 96.67; Q3 141.5) for patients with and without PDR (difference: 9.4, p=0.043).

Furthermore, a logistic analysis was carried out to explore correlations between PDR and investigated biomarkers in the study groups. In the multivariate analysis, NLR (OR=2.01, [1.29; 3.14], p= 0.0019) and diabetic nephropathy (OR=3.84, [1.23; 11.98], p= 0.0203) were associated with higher rates of PDR (Table 3).

**Table 3:** Multivariate regression analysis for dependent variables for PDR *p<0.05; **p<0.01; ***p<0.001; ****p<0.0001 PDR: proliferative DR; NLR: neutrophil-to-lymphocyte ratio

Variable	Odds ratio [confidence interval]	p-value
Intercept	0.0567 [0.014; 0.229]	<0.0001 ****
Diabetic nephropathy	3.84 [1.23; 11.98]	0.0203 *
NLR	2.01 [1.29; 3.14]	0.00192 **

The PLR (OR=1.01, [1.0; 1.02], p= 0.0062) was associated with higher rates of PDR, but it did not bring any additional value to the model. In the multivariate analysis, HbA1C (OR=1.17, [0.77; 1.77], p= 0.4719), creatinine (OR=2.64, [0.72; 9.64], p= 0.1414), neutrophils (OR=1.3, [0.95; 1.78], p= 0.0952), and thrombocytes (OR=1.0, [0.99; 1.01], p= 0.812) were not associated with the rate of PDR.

## Discussion

The pathophysiology of DR is complex and multifactorial, involving chronic hyperglycemia, oxidative stress, and inflammation. Chronic low-grade inflammation is a key factor in the pathophysiology of DR, with systemic inflammation causing disruption of the blood-retinal barrier, endothelial dysfunction, increased adhesion molecules, and a higher influx of inflammatory cells in the retina [12,13]. Increased levels of inflammation and immune response lead to the upregulation of pro-inflammatory cytokines and chemokines, such as interleukin-1β (IL-1β), tumor necrosis factor-alpha (TNF-α), and vascular endothelial growth factor (VEGF), which can exacerbate endothelial dysfunction, leukocyte infiltration, and retinal neovascularization in DR [14,15].

By evaluating these ratios in a cohort of diabetic individuals, we aim to assess their potential as non-invasive biomarkers for identifying individuals at a higher risk of developing DR and predicting its progression. A higher NLR indicates a higher level of systemic inflammation, as neutrophils are the primary mediators of the acute inflammatory response, while lymphocytes modulate the immune response. Several studies have demonstrated a positive association between elevated NLR and the presence and severity of DR, suggesting that NLR could serve as a potential biomarker for identifying patients at risk of DR and monitoring disease progression [16,17].

Platelets play a crucial role in the inflammatory process, releasing pro-inflammatory mediators and contributing to endothelial dysfunction. An increased PLR has also been associated with the presence and severity of DR, reflecting a heightened inflammatory state and increased risk of microvascular complications in patients with DM [17,18]. In our study, the PLR correlates with the DR and PDR, but to a lesser extent than the NLR.

It is important to note that both the NLR and PLR can be affected by various factors, such as acute infections, autoimmune diseases, and hematological disorders, which may influence the interpretation of these markers in DR. Additionally, pre-analytical factors, such as sample collection and handling, can impact the accuracy and reliability of CBC test results. Therefore, it is essential to consider these factors when interpreting the NLR and PLR in clinical practice [19]. The findings from this study could have significant clinical implications by providing healthcare professionals with a simple, cost-effective, and readily available tool for assessing DR risk and monitoring disease progression. Furthermore, a deeper understanding of the association between NLR, monocytes, PLR, and DR may shed light on the interplay between inflammation, immune response, and microvascular dysfunction in T2DM.

The NLR and PLR are promising biomarkers for evaluating the inflammatory status in DR. However, to maximize their clinical utility, it is essential to establish appropriate classification systems or cutoff values for the NLR and PLR about the presence and severity of DR. This section will discuss the current approaches to classifying NLR and PLR changes in DR and the challenges in defining optimal cutoff values.

Several studies have attempted to establish cutoff values for the NLR and PLR to differentiate between patients with and without DR or to stratify patients according to DR severity. These cutoff values calculated using various statistical methods, such as receiver operating characteristic (ROC) curve analysis, which determines the optimal cutoff value that maximizes sensitivity and specificity. However, the proposed cutoff values have varied considerably across studies, reflecting differences in patient populations, study designs, and statistical methods [20,21]. Moreover, the relationship between the NLR, PLR, and DR is not linear, and some studies have suggested that changes in the NLR and PLR may be more informative when analyzed as continuous variables rather than categorical variables based on arbitrary cutoff values. For instance, a higher NLR or PLR associated with an increased risk of DR or more severe DR. However, the precise risk may depend on other factors, such as glycemic control, duration of diabetes, and the presence of other microvascular or macrovascular complications [22,23].

Previously published studies found an ascending trend for the NLR and PLR associated with the presence and the severity of DR [24-30]. However, the mean NLR values varied widely from 1.99±1.03 to 2.4±1.9 in T2DM patients without DR and from 1.95±0.17 to 4.4±2.7 in those who presented signs of DR [26-29]. Moreover, Hu et al. [30] found that higher NLR values were associated with poor response to anti-VEGF therapy in patients with diabetic macular edema. There are fewer data regarding the PLR values in T2DM patients with or without DR. Akdogan et al. [27] found a mean value of 140±87 when diabetic microvascular changes were present, compared to 116±16 in no DR group, while Zeng et al. [31] found a mean value of 137.02 versus 105.34 in patients with and without DR, respectively. Age, sex ratio, BMI, race, diabetic control, and other associated pathologies may explain these differences encountered in clinical studies. This significant variation is the main limitation yet to overcome before using this important biomarker in clinical practice.

One approach to addressing the challenges in classifying NLR and PLR changes in DR integrates these biomarkers into a comprehensive risk prediction model together with other relevant clinical and laboratory parameters. However, further research is needed to develop and validate such models in diverse patient populations.

Our study was not devoid of limitations. The relatively small sample size (90 patients), selected from a single center, potentially affected the stability of our statistical estimates and the performance of our statistical analysis. Ideally, larger datasets are needed to validate and extend these findings. The cross-sectional, retrospective nature of the study design was a limitation of this study.

## Conclusions

The NLR and PLR emerged as promising inflammatory markers in DR, with potential roles in risk stratification, disease monitoring, and therapeutic decision-making. The classification of NLR and PLR changes in DR remains an area of ongoing research. While several studies have proposed various cutoff values for the NLR and PLR about DR, the optimal approach to classifying these biomarkers may involve integrating them into a comprehensive risk prediction model with other relevant factors. Future research should focus on developing and validating such models to enhance the clinical utility of the NLR and PLR in the assessment and management of DR.
